# Metabolic Dynamics and Prediction of Gestational Age and Time to Delivery in Pregnant Women

**DOI:** 10.1016/j.cell.2020.05.002

**Published:** 2020-06-25

**Authors:** Liang Liang, Marie-Louise Hee Rasmussen, Brian Piening, Xiaotao Shen, Songjie Chen, Hannes Röst, John K. Snyder, Robert Tibshirani, Line Skotte, Norman CY. Lee, Kévin Contrepois, Bjarke Feenstra, Hanyah Zackriah, Michael Snyder, Mads Melbye

**Affiliations:** 1 Department of Genetics, Stanford University School of Medicine, Stanford, CA 94305, USA; 2 Department of Epidemiology Research, Statens Serum Institut, Copenhagen, 2300, Denmark; 3 Department of Chemistry and the Chemical Instrumentation Center, Boston University, Boston, Massachusetts 02215, USA; 4 Department of Statistics and Biomedical Data Science, Stanford University, Stanford, CA 94305, USA; 5 Department of Molecular and Cell Biology, University of California, Berkeley, Berkeley, CA 94720, USA; 6 Department of Clinical Medicine, University of Copenhagen, Copenhagen, 2200, Denmark; 7 Department of Medicine, Stanford University School of Medicine, Stanford, CA 94305, USA

**Keywords:** human pregnancy, metabolic clock, metabolomics, longitudinal profiling, metabolic pathways, machine learning, gestational age, delivery prediction

## Abstract

Metabolism during pregnancy is a dynamic and precisely programmed process, the failure of which can bring devastating consequences to the mother and fetus. To define a high-resolution temporal profile of metabolites during healthy pregnancy, we analyzed the untargeted metabolome of 784 weekly blood samples from 30 pregnant women. Broad changes and a highly choreographed profile were revealed: 4,995 metabolic features (of 9,651 total), 460 annotated compounds (of 687 total), and 34 human metabolic pathways (of 48 total) were significantly changed during pregnancy. Using linear models, we built a metabolic clock with five metabolites that time gestational age in high accordance with ultrasound (R = 0.92). Furthermore, two to three metabolites can identify when labor occurs (time to delivery within two, four, and eight weeks, AUROC ≥ 0.85). Our study represents a weekly characterization of the human pregnancy metabolome, providing a high-resolution landscape for understanding pregnancy with potential clinical utilities.

## Highlights

• Weekly metabolome of maternal blood changes dynamically through healthy pregnancy
• A metabolic clock of five blood metabolites accurately predicts gestational age
• Two to three metabolites identify labor onset within two, four, and eight weeks
• Women with metabolic clocks that outpaced ultrasound evaluation tend to deliver earlier

## Graphical Abstract

**Figure:**
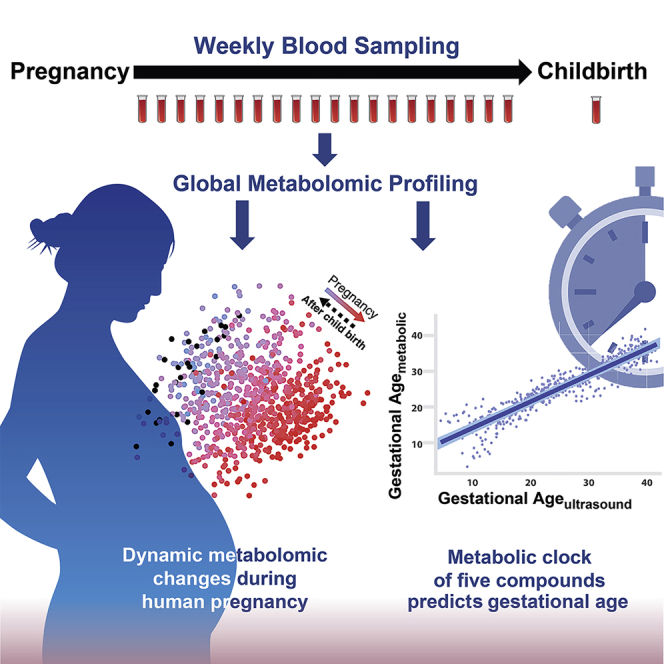

## Teaser

Identification of blood metabolites in pregnant women that can accurately predict gestational age and provide insights into pregnancy variations undetected by ultrasound.

## Introduction

Pregnancy is one of the most critical periods for mother and child (Alkema et al., 2016, Say et al., 2014). It involves a tremendous flow of physiological changes and metabolic adaptations week by week, and even small deviations from the norm might have detrimental consequences at different pregnancy stages. For example, approximately 20% of all pregnancies end in miscarriage (< 20 weeks), and around 10% end in preterm birth (< 37 weeks) (Blencowe et al., 2013, Wang et al., 2003). The latter is the leading cause of global neonatal morbidity and mortality (Blencowe et al., 2013). Of 200 million annual pregnancies, 300,000 pregnancy- and birth-related maternal deaths and 7 million perinatal deaths occur worldwide (GBD 2013 Mortality and Causes of Death Collaborators, 2015, Sedgh et al., 2014). With a better understanding of how pregnancy is regulated, even small improvements in obstetric health care can enhance the well-being of many women and children.

An accurate estimation of the timing of pregnancy and birth is important for many clinical decisions in obstetrics, including determination of preterm birth and related treatment regimens (Committee on Obstetric Practice, the American Institute of Ultrasound in Medicine, and the Society for Maternal-Fetal Medicine, 2017). The current clinical method of determining the gestational age and due date is based on information about the last menstruation date, which can be imprecise, or ultrasound imaging, which depends on accessibility at early pregnancy (Committee on Obstetric Practice, the American Institute of Ultrasound in Medicine, and the Society for Maternal-Fetal Medicine, 2017). Missing the time window is common even in developed countries: in the United States, approximately 900,000 pregnancies annually do not have a prenatal visit before the second or third trimester (Martin et al., 2018).

The maternal circulatory system connects with the fetal circulatory system through the placenta, carrying bioactive molecules and biomarkers such as steroid hormones, micronutrients, and circulating nucleic acids, whose concentrations alter as gestation progresses (King, 2000, Koh et al., 2014, Tulchinsky et al., 1972, Wang et al., 2016). Recent work on cell-free RNA suggests that markers in maternal blood can be used to estimate gestational age, but sequencing can be expensive and time-consuming, and the accuracy, at present, is not ideal (Ngo et al., 2018). Therefore, a more accurate and cost-effective method for estimating gestational age and delivery time, possibly using blood metabolites, is needed. In addition, current clinical tests often only focus on a few markers, whereas research covering more molecules often examines the profiles at one or a few time points during pregnancy (Bahado-Singh et al., 2012, Chan et al., 2003, Dudzik et al., 2014, Gagnon et al., 2008, Kenny et al., 2010, Koh et al., 2014, López-Hernández et al., 2019, Romero et al., 2010, Sachse et al., 2012, Soldin et al., 2005). Thus, a high-resolution landscape of pregnancy-related metabolites during healthy pregnancy and the postpartum period is still poorly understood.

Here, we use untargeted metabolomics (Kaddurah-Daouk et al., 2008) to systematically profile blood metabolites throughout pregnancy with weekly sampling of maternal blood. The study identified a large number of pregnancy-related metabolites and metabolic pathways offering a comprehensive view of the metabolite changes during healthy pregnancy and the postpartum period. Leveraging the high-resolution datasets, we built a metabolic clock that not only predicts gestational age in high accordance with the first-trimester ultrasound, the clinical gold standard, but also recovers personal pregnancy variations undetected by ultrasound but capable of affecting delivery time.

## Results

### Danish Pregnancy Cohort: A Study of Normal Pregnancy with High-Density Sampling

**Figure 1:**
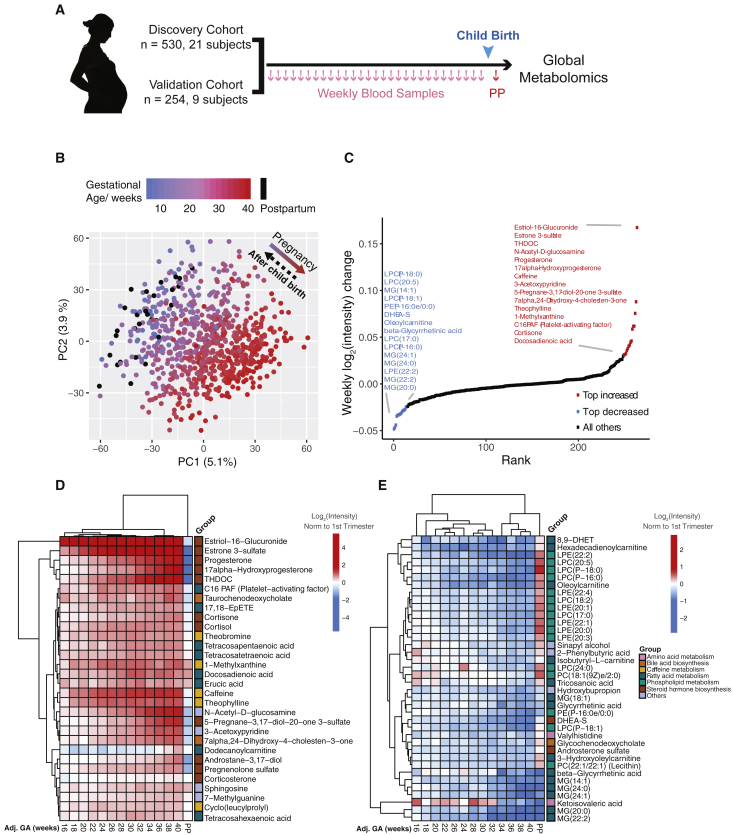
Untargeted Metabolomics Cluster the Weekly Plasma Samples Precisely According to Gestational Age (A) Sampling scheme. Note that validation cohort refers to test set 1 in Table 1. (B) Principal component analysis (PCA) distributed individual samples according to pregnancy stages (based on 9,651 features). The two PCs explaining the largest part of the variation are shown. (C) Plot shows the top 15 increased (red) and decreased (blue) metabolites (with MSI level 1 or 2 identification) in pregnancy. (D and E) Heatmap displays the metabolite signal intensity averaged across individuals, showing the top 68 altered metabolites (D) increased and (E) decreased by the end of pregnancy. Abbreviations are as follows: PP, postpartum. The gestational ages (GAs) were calculated by scaling delivery events to 40 weeks. The week order, which mostly coincides with the actual order, was ordered by hierarchical clustering on the basis of Manhattan distances. The intensities averaged before 14 weeks of all women were used as the baseline. See also Figure S1 and Table S1.

**Figure S1:**
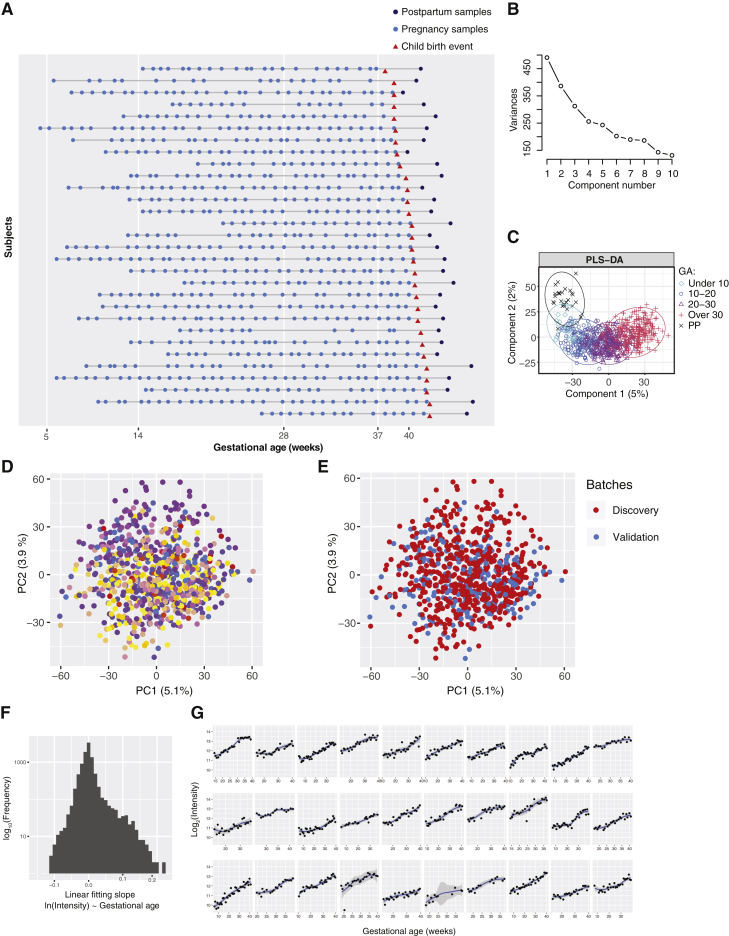
Untargeted Metabolomics for Longitudinal Pregnancy Samples, Related to Figure 1 (A) High-density longitudinal sampling of pregnancies. (B) The Scree plot of the principal component analysis. (C) The PLS-DA result according to the categories of gestational age. GA: gestational age; PP: postpartum. (D and E) Principal component analysis based on all 9,651 features shows that the samples do not separate according to the 30 subjects (D) samples from individual subjects are represented by different colors or experimental batches of Discovery and Validation (Test Set 1) analyzed across two different years (E) samples of the discovery cohort are presented in red; samples of the validation cohort (Test Set 1) are presented in blue. (F) Histogram shows the distribution of slopes in the linear fitting model of the 9,651 features (intensities against the gestational ages). (G) For each of the 30 women, the intensities of an example metabolic feature are shown over the course of gestation, which reveals consistent increases in abundance according to gestational age among 30 subjects, despite individual differences.

To capture the highly dynamic pregnancy process at high resolution, we established a multi-year single-center Danish normal pregnancy cohort and a design of high-density blood sampling. Consenting female participants submitted weekly blood draws beginning in week 5 of pregnancy and ending in the postpartum period. A total of 30 women with weekly blood samples were assigned to a discovery (N = 21) and a validation (test set 1, N = 9) cohort (Table 1; Figures 1A and S1A). The samples were analyzed in two separate years. In addition, another separate set of women (N = 8) was included as the secondary validation cohort. These samples were analyzed independently three years apart from the discovery cohort (test set 2) (Table 1).

**Table 1:** Demographics and Birth Characteristics of the Discovery and Validation Cohorts

	**Discovery**	**Test Set 1**	**Test Set 2**
	**N = 21**	**N = 9**	**N = 8**
**Demographics**			

Maternal age at birth, years	29.8 ± 3.1	29.7 ± 3.3	31.4 ± 1.0
**Previous births, No. (%)**			
0	13 (61.9)	6 (66.7)	4 (50.0)
1	8 (38.1)	2 (22.2)	3 (37.5)
≥ 2	0 (0)	1 (11.1)	1 (12.5)
Pre-pregnancy BMI, kg/m^2^	22.1 ± 2.9	21.2 ± 3.4	21.1 ± 1.6
**Smoking during pregnancy, No. (%)**			
Yes	0 (0)	0 (0)	1 (12.5)
No	18 (85.7)	9 (100)	6 (75.0)
Missing	3 (14.3)	0 (0)	1 (12.5)
**Alcohol during pregnancy, No. (%)**			
Yes	5 (23.8)	1 (11.1)	1 (12.5)
Average number of units per week	0.80	1.0	0.25
No	13 (61.9)	8 (88.9)	6 (75.0)
Missing	3 (14.3)	0 (0)	1 (12.5)

**Birth characteristics**			

Gestational age, days	281 ± 8.4	280.7 ± 8.3	279.3 ± 9.5
**Mode of delivery, No. (%)**			
Spontaneous vaginal birth	10 (47.6)	5 (55.6)	4 (50.0)
Induced vaginal birth	7 (33.3)	1 (11.1)	3 (37.5)
C-section before onset of labor	1 (4.8)	3 (33.3)	1 (12.5)
C-section during labor	3 (14.3)	0 (0)	0 (0)
Birth weight, grams	3,638 ± 500	3,803 ± 662	3,362 ± 493
Birth length, centimeters	52.4 ± 2	53.3 ± 2	51 ± 2.3
**Gender of child, No. (%)**			
Male	9 (42.9)	5 (55.6)	5 (62.5)
Female	12 (57.1)	4 (44.4)	3 (37.5)

Values are means (SDs) or numbers (percentages).

### Weekly Pregnancy Progression Is Precisely Ordered by Metabolites

We randomized the 784 samples from the first 30 subjects within each cohort (Discovery and test set 1), processed them by using a standardized protocol (Contrepois et al., 2015), and analyzed them by liquid chromatography-mass spectrometry (LC-MS) for untargeted metabolomics across two separate years. After quality control, data filtering, and normalization (see STAR Methods) (N = 30), we identified 9,651 metabolic features across the different samples. Of these, 4,995 features (51.7%) were altered during pregnancy and/or the postpartum period (false discovery rate [FDR] < 0.05), suggesting extensive metabolic changes occur during pregnancy. We examined the data globally with principal component analysis (PCA), in which the samples were distributed on the basis of the first two principal components according to their gestational stages (Figure 1B; Scree plot in Figure S1B and the partial least-squares discriminant analysis [PLS-DA] results in Figure S1C), regardless of individual variation and batches (Figures S1D and S1E). Interestingly, we found that metabolites with uni-directional behaviors dominated the features, and over half of them increased across pregnancy until reaching their peaks immediately before labor (Figures S1F and S1G).

To understand the potential function of pregnancy-related metabolites, we first annotated metabolic features by using an in-house library and a combined public spectral database (see details in STAR Methods). A total of 952 metabolic features were mapped to 687 compounds, which include plasma metabolites with important functions in humans. We then applied significance analysis for microarrays (SAM) to examine the correlation between the abundance of each compound and the reported gestational age of a woman at blood sampling. Among the 687 annotated compounds, 460 compounds were significantly associated with pregnancy (67.0%; FDR < 0.05, SAM). In addition, 264 compounds were identified with a metabolomics standards initiative (MSI) level 1 or 2 identification (Viant et al., 2017), among which 176 compounds (66.7%) were significantly associated with pregnancy, as determined by linear regression with gestational age (FDR < 0.05, SAM).

Our dense sampling revealed detailed temporal patterns of molecular changes. Among the top 68 metabolites (of the 176) that changed over 50% during whole pregnancy, those that increased (N = 30) included steroid hormones estriol-16-glucuronide, estrone 3-sulfate, and tetrahydrodeoxycorticosterone (THDOC). All three increased more rapidly than the well-known steroids progesterone and 17α-hydoxyprogesterone (FDR < 0.05, SAM) (Figures 1C and 1D). By contrast, the top metabolites that decreased during pregnancy (N = 38) were mostly lipids or lipid-like molecules, such as monoacylglycerides (MGs), lysophosphatidylcholines (LPC or lysoPC), and oleoylcarnitine (Figures 1C and 1E). Hierarchical clustering of the weekly samples on the basis of the top 68 altered metabolites revealed a week-order mostly consistent with the actual progression of gestational age (Table S1; Figures 1D and 1E). Intriguingly, most of these metabolite changes rapidly returned to baseline after childbirth (postpartum) (Figures 1B and 1D and 1E). Together, these results suggest a dramatic and programmed change of human blood metabolites at a system level during pregnancy.

### Metabolite Groups Altered during Pregnancy

**Figure S2:**
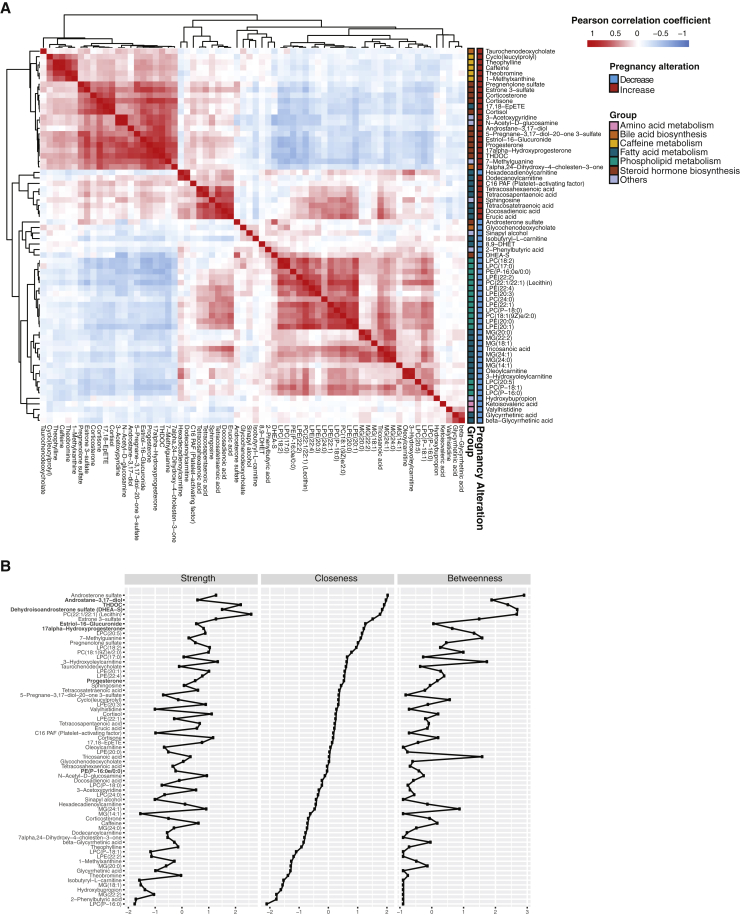
Functional Metabolite Groups Altered during Pregnancy, Related to Figure 2 (A) Correlation matrix colored by the Pearson correlation coefficient of each pair of pregnancy-related compounds across samples. (B) The strength, closeness, and betweenness of metabolites in the regularized partial correlation network indicate how important the metabolites are in the network. Metabolite names are listed on the left side ranked by the closeness, with the names of the seven compounds in the prediction models of Figure 4 and Figure 5 (bold).

**Figure 2:**
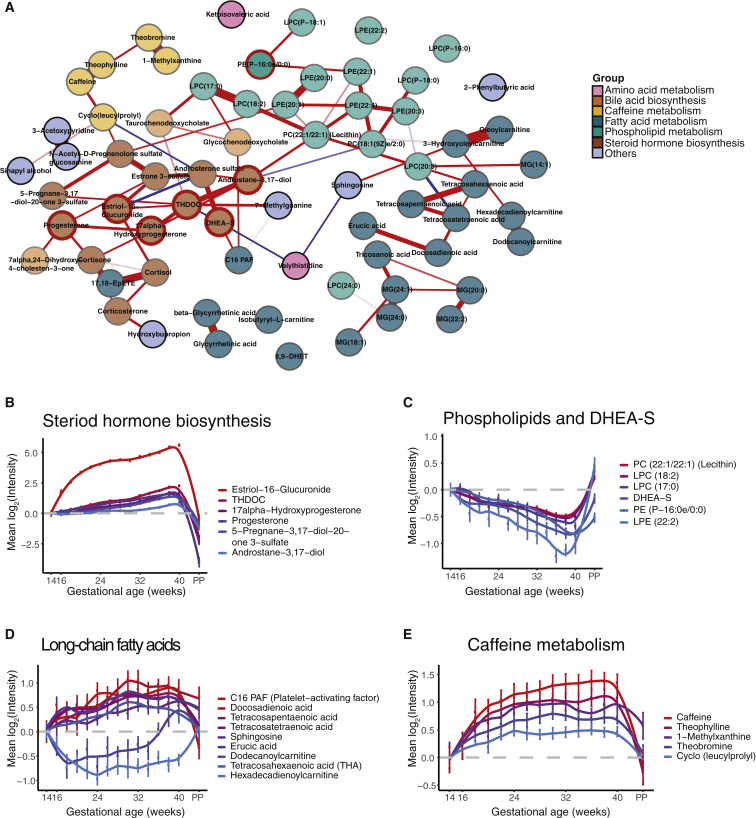
Functional Metabolite Groups Altered during Pregnancy (A) Regularized partial correlation network of top altered compounds in pregnancy. Here, each node represents a compound, and each edge represents the strength of partial correlation between two compounds after conditioning on all other compounds in the datasets. Edge weights represent the partial correlation coefficients. Note that the seven nodes with red circles with central positions were also the predictors in the models of Figures 4 and 5. (B–E) The average levels of the metabolite changes against the gestational progression in the clusters of steroid hormone biosynthesis (B), phospholipids and DHEA-S (C), long-chain fatty acids (D), and caffeine metabolism (E). The intensities were normalized to the baseline, which was defined by averaging all samples before 14 weeks. The standard errors, derived from 30 subjects, are shown. The GAs were standardized by scaling delivery events to 40 weeks. Abbreviation is as follows: PP, postpartum. Note that the y axis scale is much larger for steroids than for other compounds. See also Figure S2.

To detect the functional groups of metabolites that change during pregnancy, we performed correlation analysis on the temporal intensity profiles of the top 68 pregnancy-related compounds mentioned above. In Figure S2, metabolites that were significantly increased or decreased tended to cluster together. Using existing structural and biological information, we first categorized the top changing compounds in pregnancy into seven groups. Interestingly, compounds of the same groups tended to cluster together in the correlation matrix. On the basis of the correlation relationship, we constructed a regularized partial correlation network using all pregnancy-related compounds to explore the potential regulatory relationships (Figures 2A and S2B). The topology of the network indicates that different metabolite groups occupied different positions; dense interactions occurred between both inter- and intra- metabolite groups with the densest interactions between central steroid hormones (Figure 2A). These findings highlight that even though the amount of each compound dynamically changes during pregnancy, a highly coordinated metabolite regulatory network underlies the pregnancy process.

We next examined the main clusters that were present in the correlation analysis. Three main clusters emerged from the hierarchical clustering of metabolites (Figure S2A), with a steroid cluster (e.g., antrostane-3,17-diol, estriol-16-glucuronide, progesterone, 17α-hydroxyprogesterone, and THDOC) sitting between the large clusters of lipids and non-lipid molecules. Compared with the other steroids in this cluster that slowly but steadily increased throughout pregnancy, estriol-16-glucuronide exhibited a rapid increase before week 24, (Figures 1D and 2B). Nearly all upregulated metabolites positively correlated with this cluster of steroids, whereas all downregulated metabolites negatively correlated with this cluster (Figure S2A). This result suggests that different steroid hormones might regulate global metabolome dynamics during pregnancy.

Within the lipid cluster, intra-correlation was relatively high. The largest cluster was composed of LysoPCs (Figures 2A and S2A), a class of phospholipids. LysoPCs gradually decreased during pregnancy and increased after childbirth in a pattern that highly correlates with the steroid dehydroepiandrosterone sulfate (DHEA-S) (Figure 2C). LysoPCs are bioactive pro-inflammatory lipids that have been linked with organismal oxidative stress and inflammation (Sevastou et al., 2013). The second-largest cluster of lipids included several free fatty acids that were highly correlated within the cluster (Figures 2A and S2A). Long-chain fatty acids showed intricate dynamics in their amounts revealed by the dense sampling. Hexadecadienoylcarnitine and tetracosahexaenoic acid (THA) decreased at the beginning of pregnancy, followed by waves of increased amounts similar to other fatty acids in the late second and third trimesters (Figure 2D). After childbirth, the amounts of most long-chain fatty acids decreased, except for hexadecadienoylcarnitine (Figure 2D).

Within the non-lipid cluster, one sub-cluster included five highly correlated metabolites belonging to the same caffeine metabolism pathway (Figures 2A and S2A). All five metabolites were consistently elevated during pregnancy, and caffeine reached a concentration three times higher at the end of pregnancy than at the beginning (Figure 2E). This elevation might be due to a slower caffeine metabolism in pregnant women rather than an increase in coffee intake (Knutti et al., 1981). Overall, among the 68 top-altered metabolites in pregnancy, functional metabolite groups (e.g., steroids, LysoPCs, fatty acids, and caffeine metabolites) were altered in an orchestrated manner during pregnancy, and individual compounds within each group showed inter-correlation to each other (Figure 2A).

### Orchestrated Metabolome Reconfigurations Span Multiple Pathways during Pregnancy

**Figure 3:**
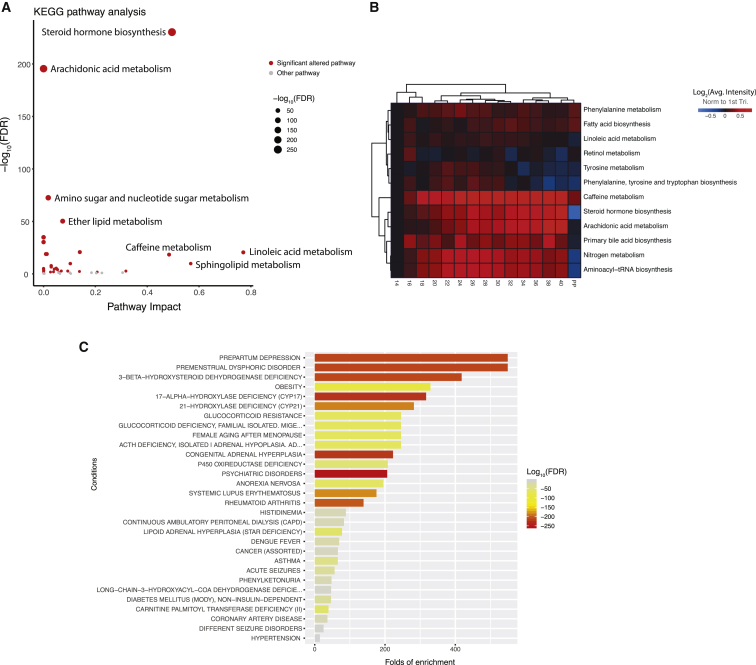
System-Wide Reconfiguration of Metabolic Pathways during Pregnancy (A) Metabolic pathways undergoing significant changes during pregnancy. Red dots denote pregnancy-related pathways with FDR < 0.05, which were further analyzed in (B). The topological pathway effects were quantified by using published methods (Xia and Wishart, 2010a). (B) Heatmap shows the temporal changes of pregnancy-related pathway activities during pregnancy and postpartum (PP). To quantify pathway activity, the average intensity of metabolites in each pathway at each time window was calculated. Note that although some pathways contained mainly the metabolites increasing or decreasing during pregnancy, many pregnancy-related pathways contained both metabolites increasing and decreasing. Thus, their average values would not show large changes in the heatmap. For each pathway, the average values from samples earlier than 14 weeks (marked as week 14) were used as the baseline. (C) Human disease states that correlated with pregnancy-related metabolites on the basis of published metabolomics data (Chong et al., 2018). See also Figure S3.

**Figure S3:**
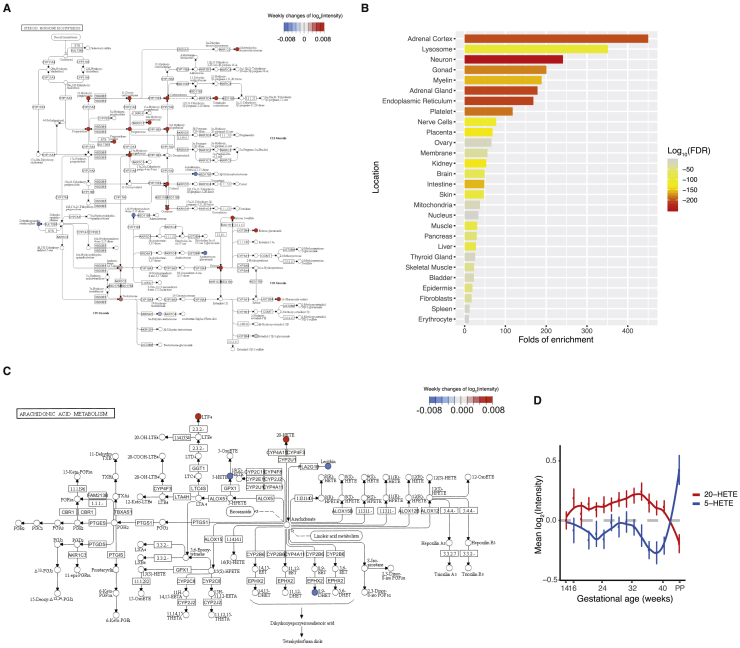
Pregnancy-Related Metabolic Pathways and Metabolite Origin Analysis, Related to Figure 3 (A) Steroid hormone biosynthesis pathway, with metabolite increases (in red) or decreases (in blue) over the course of gestation. (B) Numerous metabolites in plasma that were altered during pregnancy can be traced back to organs by metabolite set enrichment analysis (MSEA). (C) Arachidonic acid metabolism pathway, with metabolite increases (in red) or decreases (in blue) over the course of gestation. (D) The average levels of the 20-HETE and 5-HETE changes against the gestational progression. The intensities were normalized to the baseline, which was defined by averaging all samples before 14 weeks. The standard errors, derived from 30 subjects, are shown. The gestational ages were adjusted by scaling delivery events to 40 weeks. PP, postpartum.

Next, we longitudinally examined the global pathway changes of all 687 annotated compounds during normal pregnancy. Among the 48 mapped Kyoto Encyclopedia of Genes and Genomes (KEGG) pathways, 34 showed significant changes (70.8%, adjusted FDR < 0.05, global test) (Goeman et al., 2004, Xia and Wishart, 2010a) through metaboanalystR (Figure 3A) (Chong et al., 2018), suggesting large-scale pathway changes of metabolism in pregnancy. To quantify the pathway activities through gestational age, we calculated the average intensity of metabolites in the pathways (Figure 3B; see STAR Methods). Among the top altered pathways (Figure 3A), steroid hormone biosynthesis showed elevated activity precisely timed to gestation, peaking before the end of pregnancy and then declining sharply shortly after delivery (Figure 3B). Along with the essential roles of steroid hormones in maintaining pregnancy and later inducing parturition (Mendelson, 2009), we observed an orchestrated elevation of many components centered on progesterone, including some less well-characterized hormones (Figure S3A). Consistent with known sources of pregnancy metabolites (e.g., hormones) (Maltepe and Fisher, 2015), metabolite set enrichment analysis (MSEA) (Xia and Wishart, 2010b) revealed that the adrenal cortex, gonad, and placenta were among the top origins of pregnancy-related metabolites (Figure S3B). The ability to recognize many well-known and less-characterized steroid hormone changes across pregnancy validates our approach.

In addition to the steroid pathway, we observed a dynamic pattern of metabolite changes with pregnancy in other pathways, such as the arachidonic acid metabolism pathway (Figures 3A, 3B, and S3C). We observed 20-HETE amounts increased until week 34; 20-HETE is potentially linked to the regulation of blood pressure and renal function during pregnancy (Wang et al., 2002, Wu et al., 2014) (Figures S3C and S3D). By contrast, 5-HETE amounts generally decreased during pregnancy, potentially associated with its regulation of the uterus (Figures S3C and S3D) (Edwin et al., 1997, Pearson et al., 2010). Thus, beyond energy metabolism and hormones, a system-wide reconfiguration of the metabolome occurs as the mother adapts to pregnancy. In addition, based on MSEA analysis, many pregnancy-related metabolites are implicated in human disease states, including obesity and prepartum depression (Figure 3C) (Xia and Wishart, 2010b).

### The Metabolic Clock of Normal Pregnancy Identified by Machine Learning

**Figure 4:**
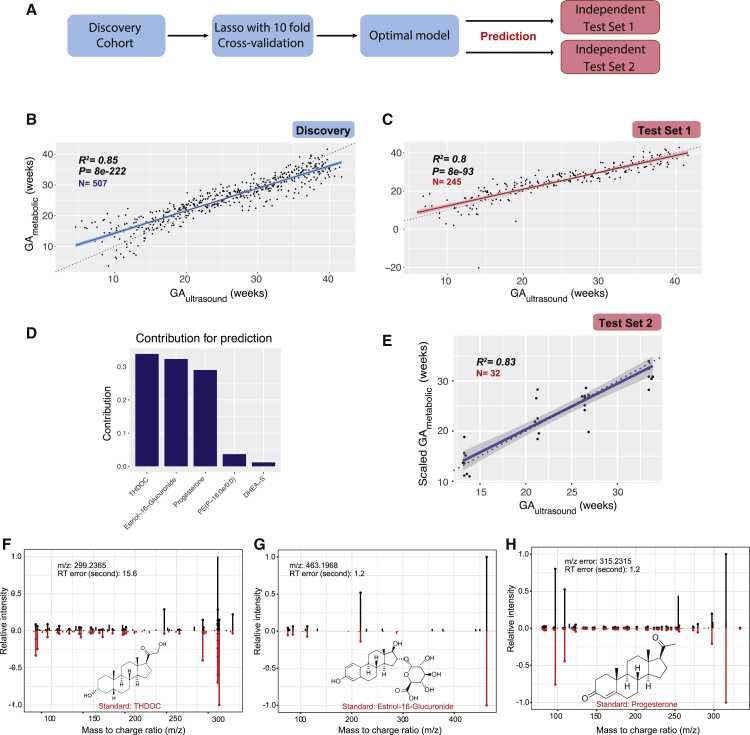
Metabolic Clock Of Pregnancy: Five Metabolites Selected by Machine Learning Can Accurately Predict the Timing of Normal Pregnancy Progression in Both a Discovery and Two Validation Cohorts (A) Design of the analytical pipeline. (B and C) Gestational age (GA) predicted by the linear model consisting of five identified metabolites (GA_metabolic_, y axis) highly correlates with clinical values determined by the standard of care (by first-trimester ultrasound [GA_ultrasound_] x axis) in the Discovery (B) and the validation cohort (test set 1) (C). Note that two samples presented as outliers in the validation cohort, possibly because of occasional mass-spectrometry signal instability in given samples. The 95% confidence interval for the linear regression is represented by the gray area. (D) Contribution of the five metabolites to the gestational age prediction model. (E) Gestational age predicted by the five metabolites (GA_metabolic_, y axis, scaled) correlates with clinical values determined by the standard of care (by first-trimester ultrasound [GA_ultrasound_] x axis) in the test set 2 cohort. The 95% confidence interval for the linear regression is represented by the gray area. (F–H) Confirmation of the metabolites predicting gestational age in the metabolic clock model by standard compounds, THDOC (F), estriol-16-glucuronide (G), and progesterone (H) (see two additional compounds PE(P-16:0e/0:0) and DHEA-S in Figures S4E and S4F). Measured MS/MS spectral fragmentation profiles (top, in black) matching chemical standards (bottom, in red). Note that the discovery results were from the 10-fold CV to avoid over-fitting (see STAR Methods). See also Figures S4 and S5 and Tables S2–S4.

We next determined whether we can build a metabolic clock based on the high-resolution profile to predict gestational age for individual plasma samples. In the discovery cohort (samples n = 507, subjects N = 21), we applied feature selection (lasso [least absolute shrinkage and selection operator]) with all 9,651 features to build the linear regression model that shows optimal cross-validation performance for predicting a given phenotype in this cohort. We then ran the validation cohort data (test set 1, samples n = 245, subjects N = 9) through the model established in the discovery cohort to measure the independent performance of our model (Figure 4A; see STAR Methods).

**Figure S4:**
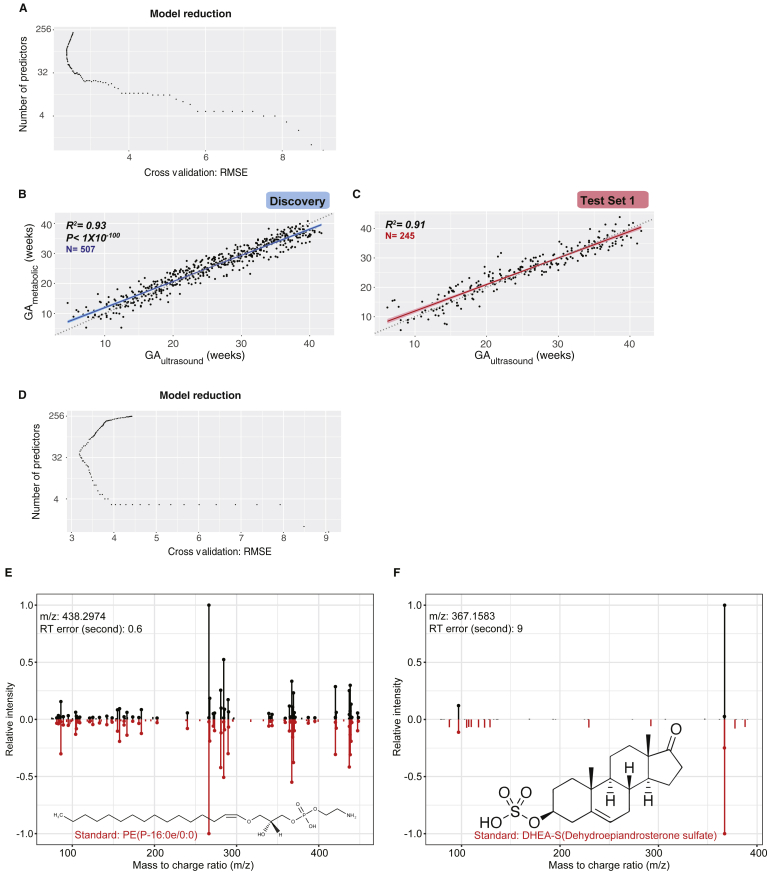
Metabolites Predict Gestational Age in Machine-Learning Models, Related to Figure 4 (A) Feature selection for predicting gestational age (GA) using metabolomic features. (B and C) GA predicted by metabolic features (GA_metabolic_, y axis) highly correlates with clinical values determined by standard of care (by first-trimester ultrasound, GA_ultrasound_, x axis) in the Discovery (B) and the validation cohort (Test Set 1) (C). The 95% confidence interval for the linear regression is represented by the gray area. (D) Feature selection for predicting GA using identified metabolites. (E and F) Measured MS/MS fragmentation profiles (upper) matching of PE(P-16:0e/0:0) (E) and DHEA-S (F) with the MS/MS of standard compounds (lower). GA, gestational age.

We first tested whether the metabolome change can quantitatively determine the gestational age in normal pregnant women. Feature selection in the discovery cohort yielded a linear model that included 42 metabolic features (Figure S4A; Table S2). In the cross-validation test of 507 samples in the discovery cohort, the metabolic model predicted gestational age in weeks (GA_metabolic_) that correlated with gestational age estimated by the first-trimester ultrasound (GA_ultrasound_, in compliance with the clinical standard of care) with a Pearson correlation coefficient (R) of 0.96 (R^2^ = 0.93, p < 1 X 10^−100^, root mean squared error [RMSE]= 2.49) (Figure S4B). In the independent-validation cohort, the model yielded a similar R of 0.95 (R^2^ = 0.91, p < 1 X 10^−100^, RMSE = 2.76, test set 1) (Figure S4C). This indicates metabolic features can accurately predict the gestational age on the basis of a blood sample from a pregnant woman.

For potential clinical use, we next tested whether we can use the annotated compounds in blood to predict the gestational age in pregnant women. We performed feature selection in discovery cohort by using the 264 level 1 and level 2 compounds identified in the Human Metabolome Database (HMDB) in the discovery cohort (Table S3). This yielded a linear model including five compounds (Figures S4D and 4D) that together are highly predictive. We first evaluated the performance of the model in a 10-fold cross-validation (CV) test in the discovery cohort, in which samples were distributed into folds by subject instead of by sample to prevent person-specific information cross-over between the training folds and the test fold. In the CV test, the metabolic-clock model produced a result (GA_metabolic_) that correlated with the gestational age estimated by the first-trimester ultrasound (GA_ultrasound_) with a Pearson correlation coefficient (R) of 0.92 (R^2^ = 0.85, p = 8e−222, RMSE = 3.67) (Figure 4B). To avoid the hyperparametric selection bias, we further evaluated the performance of our model in two independent validation cohorts (test set 1 and test set 2). In test set 1, the model yielded an R of 0.89 (R^2^ = 0.80, p = 8e−93, RMSE = 4.11) (Figure 4C). The model, including four steroids and one lipid (Figure 4D), was further verified in a second independent-validation cohort of eight individuals with R of 0.91 (R^2^ = 0.83, RMSE = 3.05, samples n = 32, test set 2) (Table 1; Figure 4E). The compound identifications were confirmed by chemical standards (Figures 4F–4H, S4E, and S4F; Table S4; see STAR Methods). We noted that four of these five compounds are among the central steroid cluster forming a dense correlation network with one another (Figure 2A).

**Figure S5:**
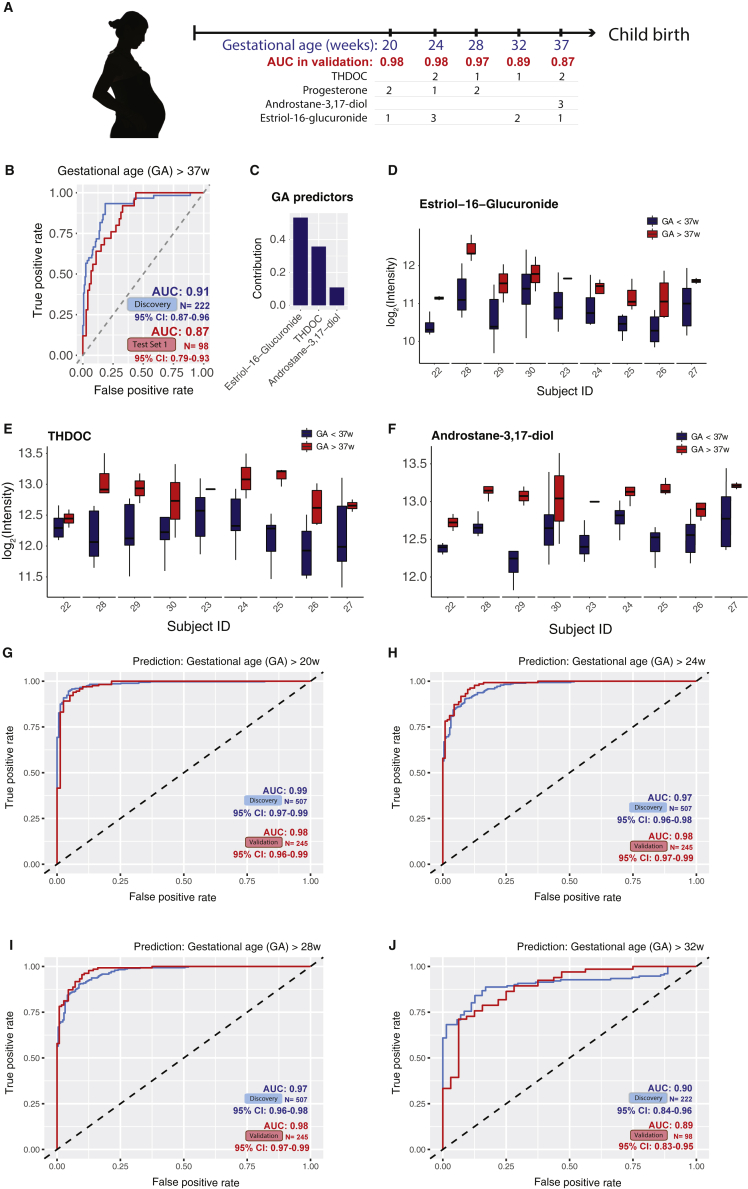
Metabolites Selected by Machine Learning Can Accurately Predict Gestational Age before or after 20, 24, 28, 32, and 37 Weeks in Both the Discovery and Validation Cohort (Test Set 1), Related to Figure 4 (A) Summary of prediction models of gestational age (GA) before or after 20, 24, 28, 32, and 37 weeks, using two to three metabolites. Note that the prediction models for 20, 24, and 28 gestational weeks were built using samples from all three trimesters and the ones for late pregnancy (32 and 37 weeks) were build using third-trimester samples. The contribution rank of each predictor in every model is listed as number 1, 2, and 3. Area under the curves (AUCs) in the validation cohort (Test Set 1) are listed. (B) The logistic regression model based on three metabolites can accurately distinguish the third-trimester plasma samples before or after 37 weeks. (C) Contribution of the three metabolites to the prediction model of gestational age before or after 37 weeks. (D) Estriol-16-Glucuronide shows intensity range separations before and after 37 weeks. (E and F) THDOC and androstane-3,17-diol show intensity range separations before/after 37 weeks. (G–J) The logistic regression models can accurately distinguish pregnancy samples before or after 20 (G) 24 (H), and 28 (I) weeks, and the third trimester plasma samples before or after 32 weeks (J). GA, gestational age.

As pregnancy progresses toward term, clinical classifications and decisions often need to be made based on the timing of pregnancy (e.g., < 37 weeks for preterm birth). Babies born before 37 weeks are considered preterm, those born before 20 weeks are considered a miscarriage, and those born before 24 weeks have low survival. Therefore, for clinical action it is important to accurately classify the gestational age by clinical cutoff points at weeks 20–37. As a proof-of-principle, we tested the potential use of the metabolome data to classify the normal pregnancy samples as before or after 20, 24, 28, 32, and 37 gestational weeks (Figure S5A). First, using only samples from the third-trimester (> 28 weeks of gestation), the time window where women were more susceptible to preterm delivery, we determined whether the identified maternal blood metabolites can distinguish the sample gestational age as before or after 37 weeks. Both the discovery and the validation prediction yielded an area under the receiver operating characteristics (AUROC) over or close to 0.90 (Figure S5B; see STAR Methods). Remarkably, the prediction model contained only three metabolites, and the abundance range of each individual metabolite separated the > 37 week samples from the < 37 week samples for all but one to two validation subjects (Figures S5C–S5F). Similarly, using samples across the whole pregnancy, we found that metabolites can also accurately distinguish pregnancy samples before or after other important gestational age cutoffs, such as 20, 24, 28, and 32 gestational weeks (Figures S5A and S5G–S5J).

### Personal Metabolic Clock of Pregnancy Linked with Timing of Delivery and Fetal Growth

**Figure 5:**
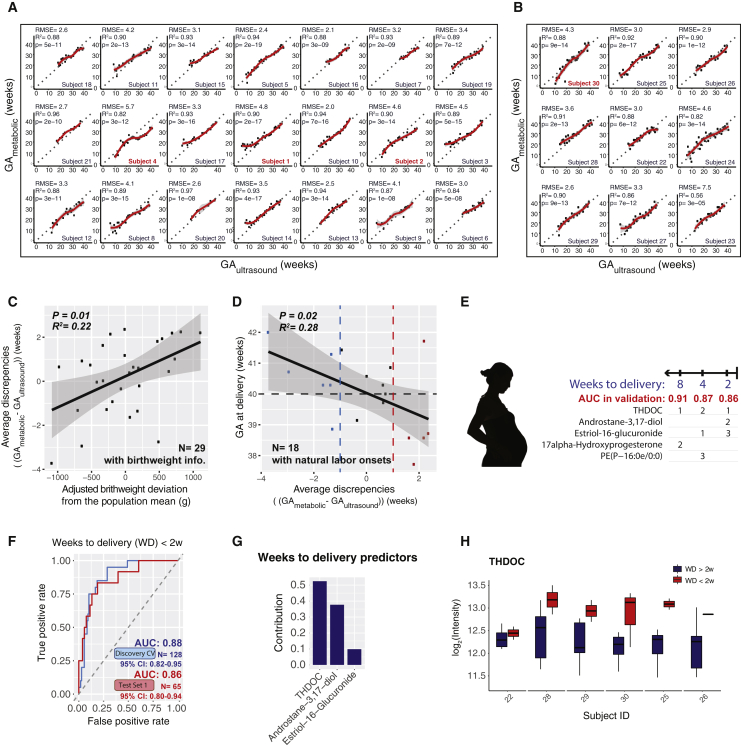
Personal Metabolic Clock of Pregnancy Linked with Timing of Delivery and Fetal Growth (A and B) Highly correlated patterns of the metabolic-clock-predicted gestational age (GA_metabolic_) of the five-metabolite model with the gestational age estimated by the first-trimester ultrasound (GA_ultrasound_) at the individual level in the cross validation (A) and test set 1 (B). Note that the outlier sample with negative prediction value in Figure 4C belonged to the last subject of the test set 1 and did not show in the current plot with the y axis scale limitation. (C) The average discrepancies between metabolic-clock-predicted gestational age and ultrasound-estimated gestational age (Δ(GA_metabolic_-GA_ultrasound_)) were significantly correlated with the fetal growth deviation from the population by person. All 29 subjects who had baby birth weight information are included here. The 95% confidence interval for the linear regression is represented by the gray area. (D) Average discrepancies between GA_metabolic_ and GA_ultrasound_ (Δ(GA_metabolic_-GA_ultrasound_)) were negatively correlated with the actual delivery weeks (by ultrasound-estimation). All 18 subjects who had natural labor onset are included here. Dashed lines marked the ultrasound estimated GA at 40 weeks (due date, black), GA_metabolic_ one week earlier than the GA_ultrasound_ (blue), and GA_metabolic_ one week later than the GA_ultrasound_ (red). The 95% confidence interval for the linear regression is represented by the gray area. (E) Summary of prediction models of 2, 4, and 8 weeks approaching delivery, using two to three metabolites. The contribution rank of each predictor in every model is listed as number 1, 2, and 3. The weeks to delivery were built using samples of the third trimester (> 28 weeks). AUCs in the validation cohort (test set 1) are listed. (F) The logistic regression model based on three metabolites can accurately identify the third-trimester plasma samples approaching the delivery (weeks to delivery [WD] < 2w; only women with natural labor onset included). (G) Contribution of the three metabolites to the prediction model of 2 weeks approaching delivery. (H) Metabolite THDOC showed abundance separations before or after 2 weeks approaching the delivery, except in one subject. See Figure S6 for other metabolites in the model. Note that the discovery results were from the 10-fold CV instead of direct fitting to avoid over-fitting. See also Figure S6 and Table S4.

**Figure S6:**
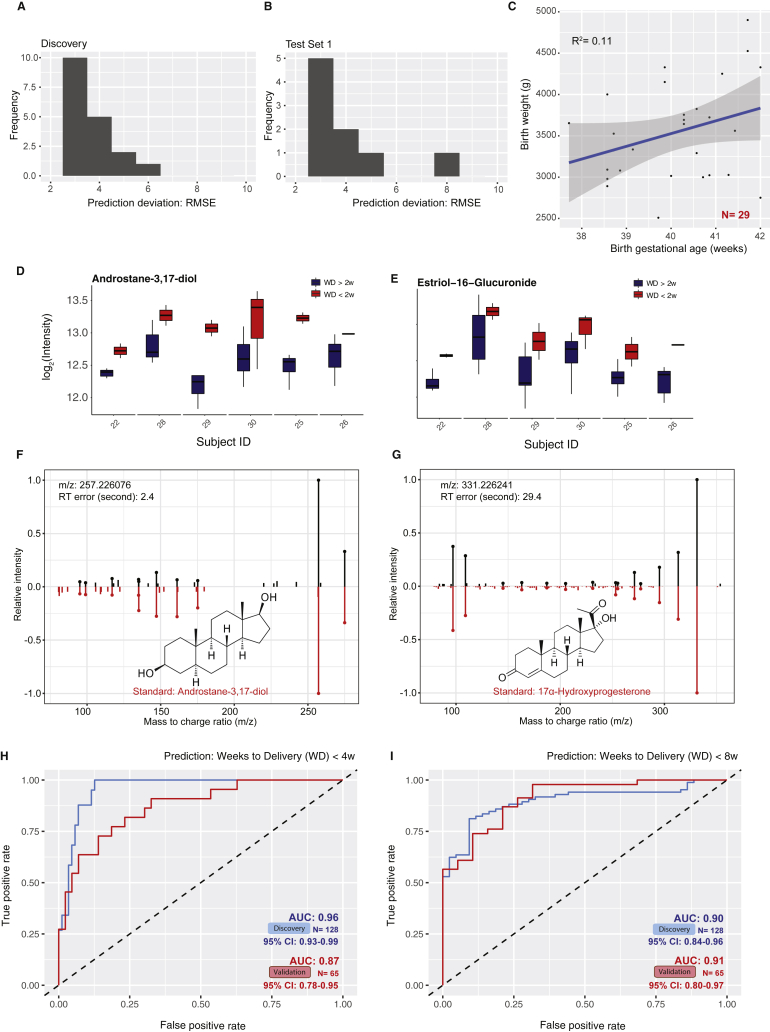
Identified Compounds Predict Gestational Age and 4 and 8 Weeks Approaching Delivery, Related to Figure 5 (A and B) Histogram shows the distribution of prediction deviation (RMSE) in the cross-validation of the discovery cohort (A) and the validation cohort (B) Test Set 1. (C) The baby birth weight shows correlation with the gestational length (gestational age at childbirth). All 29 subjects who had baby birth weight information are included here. The 95% confidence interval for the linear regression is represented by the gray area. (D and E) Androstane-3,17-diol (D) and estriol-16-Glucuronide (E) show intensity range separations before or after 2 weeks approaching the delivery. (F and G) Measured MS/MS fragmentation profiles (upper) matching of androstane-3,17-diol (F) and 17α-hydroxyprogesterone (G) with the MS/MS of standard compounds (lower). (H and I) The logistic regression models can accurately identify the third trimester plasma samples approaching delivery (weeks to delivery, WD < 4w (H), WD < 8w (I); only includes women with natural labor onset). Note that the discovery results were from the 10-fold cross-validation (CV) instead of direct fitting to avoid over-fitting.

Next, we examined the metabolic clock prediction performance in individuals. First, we noted that for most individuals, our model produced predictions consistently aligned with the gestational age estimated by the first-trimester ultrasound (Figures 5A and 5B). In both cross validations for Discovery and test set 1, the prediction deviation (measured by RMSE) in individuals centered around 3 weeks (Figures S6A and S6B). However, in each dataset, there is a small population of individuals with higher prediction deviation. When we examined these individuals (e.g., subjects 1, 2, and 4), we found the predictions were not more randomly scattered than other individuals. Rather, in the majority of them, predictions shifted away from the actual gestational ages in a portion of the pregnancy duration (Figures 5A and 5B), suggesting effects from non-random causes.

We hypothesized that some of these large prediction deviations might arise from biological causes, particularly from the maternal-fetal interaction. It is reported that the fetoplacental unit secretes hormones in conjunction with fetal growth and development (Murphy et al., 2006). Indeed, we noted that the average prediction deviation strongly correlated with adjusted infant birth weight (Figure 5C, adjusted for gestational length; see Figure S6C and STAR Methods). Thus, the overall metabolic clock tends to outpace the gestational age estimation determined by first-trimester ultrasound in mothers with a heavier fetus while being delayed in the mothers with a lighter fetus. The finding suggests that fetal growth appears to be one of the inputs read by the metabolic clock.

In addition, within the 18 women with natural labor onset (i.e., excluding women with induction before labor onset and scheduled cesarean-section), we found that the women whose overall metabolic clock of pregnancy outpaced ultrasound evaluation tended to deliver earlier, whereas a delay in metabolic clock correlated with a delayed time to child delivery compared to ultrasound estimated due date (Figure 5D). Interestingly, five out of six women (83%) with a metabolic-clock-predicted gestational age more than one week later than the ultrasound-estimated gestational age had natural labor onset after their due date (estimated by ultrasound, marked in red in Figure 5D), and four out of five women (80%) with metabolic-clock-predicted gestational age more than one week earlier than the ultrasound-estimated gestational age had natural labor onset before their due date (marked in blue in Figure 5D). These results suggest the metabolic clock of pregnancy with maternal metabolites contains information on the timing of delivery in normal pregnancy.

### Prediction for Timing of Delivery

We then tested whether the maternal blood metabolites can also predict the timing of a normal delivery event within a defined period (2, 4, and 8 weeks from delivery) approaching the labor events (in the third trimester). We first examined whether metabolites can predict a delivery within 2 weeks (weeks to delivery [WD] < 2w). To predict delivery triggered naturally without outside procedures (such as scheduled cesarean-section), we only included delivery events naturally triggered (subjects N = 18, samples n = 193). With just three metabolites, the metabolome accurately predicted an upcoming delivery event within 2 weeks in both discovery and validation cohorts with AUROC close to 0.9 (Figures 5E–5H, S6D, and S6E; see STAR Methods). Similarly, identified metabolites can also be used to predict the timing of a normal delivery event within 4 and 8 weeks (Figures 5E and S6F–S6I). Intriguingly, the panels of metabolites partially overlapped between the models, whereas the individual metabolites contributed differently to the models (Figure 5E). All of the metabolite markers were identified as steroids, except for phospholipid PE(P-16:0e/0:0), and most of them (three out of five in total) also appeared in the aforementioned metabolic clock for gestational age (Figure 5E; Table S4). These results demonstrate that we can precisely categorize critical pregnancy stages in normal subjects by using a small number of maternal blood metabolites, which can be further validated in larger and independent cohorts.

## Discussion

In this study, we performed untargeted metabolomics profiling and identified highly dynamic temporal regulation of metabolic changes in human pregnancy: more than half of the measured metabolites and metabolic pathways changed during pregnancy. We were able to detect many of the pregnancy-associated metabolite profiles revealed in previous targeted studies (Tulchinsky et al., 1972, Wang et al., 2016) (such as progesterone, 17-hydroxyprogesterone, and the linoleic acid pathway), validating our approach. At the same time, we also noted that a large portion of pregnancy-related metabolites identified in our study was less well-studied. For example, over 95% of the pregnancy-related metabolites identified in our study were not recovered from a targeted metabolic profiling study on pregnancy (Wang et al., 2016), demonstrating the power of unbiased and hypothesis-independent profiling. Among the changing metabolites, the major class that increased was steroids, including progesterone, which interacts with the hypothalamic-pituitary-adrenal axis (HPA axis) (Chrousos et al., 1998), and estriol-16-glucuronide produced by the placenta (Levitz et al., 1984). Here, the detailed differences in their temporal profiles were revealed by the weekly sampling design of the study (Figures 1D and 2B). In addition, we discovered less well-studied steroids in pregnancy, such as the neurosteroid THDOC, an allosteric modulator of the GABAA receptor that potentially affects stress and depression in human pregnancy (Hosie et al., 2006, Reddy, 2003). Intriguingly, many pregnancy-related metabolites that changed, including steroids, quickly returned to the maternal non-pregnant state after childbirth (Figures 1B and 1D and 1E). In addition, we also identified a wide variety of non-steroid hormones whose abundance altered during pregnancy progression.

These metabolite changes presumably accommodate and/or reflect important maternal biological physiology during pregnancy and fetal growth (Bispham et al., 2003, Prentice and Goldberg, 2000). For maternal nutrient metabolism, one of the decreased carnitines, oleoylcarnitine (Figure 1C), accumulates during certain metabolic conditions, including fasting (Hoppel and Genuth, 1980, Minkler et al., 2005). Also, one phosphatidylcholine that functions as a micronutrient, lecithin, increased in pregnancy, suggesting a systematic change in the maternal nutritional status during gestation. Within molecules reflecting pregnancy-related physiological changes, consistent with decreased blood pressure (Hermida et al., 1997), the antihypertensive molecule 20-HETE of the arachidonic acid metabolism pathway is elevated during pregnancy until the early third trimester, and its synthesis is regulated in a renal-specific manner (Wang et al., 2002, Wu et al., 2014). This reveals the highly dynamic temporal regulation of 20-HETE in blood pressure and kidney function during pregnancy. In contrast, compared with early pregnancy and postpartum, the amount of 5-HETE in the same pathway was generally lower in the second and third trimesters with an increasing trend right before the childbirth, consistent with previous findings that 5-HETE elevates in the uterus and amniotic fluid at the onset of human labor (Edwin et al., 1997, Pearson et al., 2010). In the developing fetus, changes in hexadecadienoylcarnitine amounts are associated with congenital heart defects (Bahado-Singh et al., 2014). Here, we revealed that the amount of hexadecadienoylcarnitine in the blood decreased continuously until week 24, then steadily increased thereafter (Figure 2D). In addition, the amount of long-chain fatty acids in maternal blood samples is associated with childhood metabolic health (Maslova et al., 2018). Here, the omega-3 fatty acid THA decreased during early pregnancy and gradually increased before childbirth (Figure 2D), suggesting gestation-related changes in the formation of docosahexaenoic acid (DHA) (Moore et al., 1995). Our findings are robust even without a requirement for prior fasting. It will be interesting to validate these findings in cohorts that have dietary information and detailed clinical measurements, to define critical “nutritional time-zones” for micronutrient amounts and further understand the metabolite changes that are important for physiologic changes across pregnancy.

Our high-density sampling scheme allowed us to study the temporal alteration of metabolite levels at weekly resolution. For example, even though many steroid metabolites were elevated during pregnancy, our profiling was able to show that there were at least two different behaviors: an early wave (such as progesterone and 17α-hydroxyprogesterone) and a second wave (such as estriol-16-glucuronide). These temporal changes of steroids across pregnancy and after childbirth are at least partially regulated by the fetoplacental unit, including both maternal adrenal gland and placenta and fetal adrenals and liver (Diczfalusy, 1953, Frandsen and Stakemann, 1961, Raeside, 2017). Further investigation into the interaction of fetal-maternal contribution will be necessary for understanding the temporal regulation of these metabolites.

Untargeted metabolome and high-density sampling enabled us to identify a broad set of high-resolution temporal profiles of metabolites during pregnancy. We hypothesized that this information might help us to understand the underlying metabolic clock that times the progression of pregnancy. We found that solely using the abundance of five compounds, without any other inputs from clinical features, we can precisely determine the gestational age of a healthy pregnant woman. The precision surpasses the recent cell-free RNA model by using maternal blood (Ngo et al., 2018). Similarly, with two to three compounds, we can categorically predict many pregnancy cutoff times with high AUC: we can determine whether a woman has reached 20, 24, 28, 32, or 37 weeks (clinical cutoffs for miscarriage, age of viability, extremely preterm, very preterm, and prematurity, respectively) into her pregnancy (Figure S5A), or whether a woman will enter into labor within the next two, four, or eight weeks (Figure 5E). The proof-of-principle study suggested that metabolome bears rich quantitative information about pregnancy progression. However, our study has its limitations. The studied population consisted of healthy Caucasian pregnant women with small variations in clinical characteristics. In the future, we need to test the models in a larger cohort with diverse ethnicities and complications. Meanwhile, targeted chemical assays need to be developed on the small panels of identified metabolite markers that were discovered by untargeted metabolomics to measure the metabolite concentration independent of batches. Intriguingly, we found the metabolic clock of pregnancy to be robust in general, but small personal deviations can be observed, most likely affected by the fetal growth (Figure 5C). Lastly, we also found that the discrepancies between metabolic timing and ultrasound suggested biological significance: the women that had advanced metabolic clock tended to deliver earlier than predicted by ultrasound, whereas a delay in metabolic clock correlated with a delayed time to child delivery (Figure 5D).

In summary, combining untargeted metabolome and high-density sampling revealed the landscape of metabolome changes during pregnancy and the postpartum period with high resolution. The data itself can serve as a resource for future research. As a proof-of-principle, we also demonstrated that the temporal abundance information of metabolome can be used to predict gestational age with high accuracy in a cohort of healthy women. There is a great need for accurate timing of pregnancy: in the US alone, 900,000 women annually missed their first-trimester ultrasound (Martin et al., 2018), currently the only accurate timing method for pregnancy (Committee on Obstetric Practice, the American Institute of Ultrasound in Medicine, and the Society for Maternal-Fetal Medicine, 2017). In low and middle-income countries, accessibility to ultrasound is even more scarce, complicating many pregnancies and fetal care down-stream (e.g., identify imminent labor, manage complications, etc.). Our study demonstrated that the development of clinical tools with a few metabolites in maternal blood to time pregnancy is promising. Testing of blood drawn from the pregnant woman would likely be limited to once or a few times to be informative and have the potential to benefit pregnant women in both developed and developing worlds.

## STAR★Methods

### Key Resources Table

**Table:** 

REAGENT or RESOURCE	SOURCE	IDENTIFIER
**Biological Samples**		

Plasma samples	This paper	N/A

**Chemicals, Peptides, and Recombinant Proteins**		

MS-grade water	Fischer Scientific	Cat#7732-18-5
MS-grade methanol	Fischer Scientific	Cat#A456-500
MS-grade acetonitrile	Fischer Scientific	Cat#A9554
MS-grade acetone	Fischer Scientific	Cat#67-64-1
MS-grade acetic acid	Sigma Aldrich	Cat#64-19-7
Progesterone	Sigma Aldrich	Cat#P-069-1ML
THDOC	Sigma Aldrich	Cat#P2016-5MG
Estriol-16-glucuronide	Sigma Aldrich	Cat#E1877-10MG
DHEA-S	Sigma Aldrich	Cat#D-066-1ML
PE(P-16:0e/0:0)	Avanti Polar Lipids	Cat#852470
Androstane-3,17-diol	Sigma Aldrich	Cat#A7755-100MG
17α-Hydroxyprogesterone	Sigma Aldrich	Cat#H-085-1ML

**Deposited Data**		

Raw data	This paper	https://www.metabolomicsworkbench.org; Project ID PR000918; Project https://doi.org/10.21228/M81H58.

**Software and Algorithms**		

Progenesis QI Software	Nonlinear Dynamics	http://www.nonlinear.com/progenesis/qi/
ProteoWizard version 3.0.19095-938eda31a	Chambers et al., 2012	http://proteowizard.sourceforge.net
the k-Nearest Neighbor algorithm	Altman, 1992	N/A
Forward Dot–Product algorithm	Stein and Scott, 1994	N/A
MetDNA	Shen et al., 2019	http://metdna.zhulab.cn/
MetaboAnalystR	Chong et al., 2018; Xia and Wishart, 2010a; Xia and Wishart, 2010b	https://github.com/xia-lab/MetaboAnalystR
R	R Core Team	https://www.R-project.org
MS/MS identification pipeline	This paper	https://jaspershen.github.io/metID/index.html

**Other**		

Zorbax SB columns (2.1 X 50mm, 1.8 Micron, 600 Bar)	Agilent Technologies	827700-914

### Resource Availability

#### Lead Contact

Further information and requests for resources and reagents should be directed to and will be fulfilled by the Lead Contact, Mike Snyder (mpsnyder@stanford.edu).

#### Materials Availability

This study did not generate new unique reagents.

#### Data and Code Availability

Original data have been deposited to the NIH Common Fund’s National Metabolomics Data Repository (NMDR) website (supported by NIH grant U2C-DK119886), the Metabolomics Workbench https://www.metabolomicsworkbench.org, Project ID PR000918. https://doi.org/10.21228/M81H58.

The code for the MS/MS identification pipeline used is available on github at https://jaspershen.github.io/metID/index.html https://github.com/jaspershen/metID.

### Experimental Model and Subject Details

#### Pregnancy cohort

We recruited pregnant women through family doctors and advertisements (Danish IRB number H-3-2014-004). At enrollment, all women were screened to ensure that they were healthy at baseline, without chronic conditions, and without medication intake of any kind (ages 23 to 36 at giving birth). From each woman, non-fasting blood samples were collected weekly during pregnancy and one sample was collected after pregnancy (2x9 mL EDTA tube and 1xPaxGene RNA tube).

### Method Details

#### Plasma sample preparation

Within Discovery and Test Set 1 cohorts, 784 normal pregnancy samples from 30 women were completely randomized and analyzed in 12 batches across two years. The 32 normal pregnancy samples in Test Set 2 were randomized and analyzed three years later. Plasma was prepared from whole blood treated with anti-clot EDTA, aliquoted, and stored at −80°C. Plasma (200 μL) was treated with four volumes (800 μL) of an acetone:acetonitrile:methanol (1:1:1, v/v) solvent mixture with internal standards (i.e., Acetyl-d3-carnitine, Phenylalanine-3,3-d2, Tiapride, Trazodone, Reserpine, Phytosphingosine, and Chlorpromazine), mixed for 15 min at 4°C, and incubated at −20°C for 2 h to allow for protein precipitation. The supernatant was collected after centrifugation at 10,000 rpm for 10 min at 4°C and evaporated under nitrogen to dryness (Biotage Turbovap). The dry extracts were reconstituted with 200 μL 1:1 methanol:water before analysis. A quality control (QC) sample was generated by pooling all the plasma samples from 10 women and injected between every 10–15 sample injections to monitor the consistency of the retention time and the signal intensity. The QC sample was also diluted two, four, and eight times to determine the linear-dilution effect of metabolic features.

#### Chemical materials for untargeted metabolomics

MS-grade water (7732-18-5), methanol (A456-500), acetonitrile (A9554), and acetone (67-64-1) were purchased from Fischer Scientific (Morris Plains, NJ, USA). MS-grade acetic acid (64-19-7) was purchased from Sigma Aldrich (St. Louis, MO, USA). Analytical grade chemical standards were purchased [Progesterone (Sigma-Aldrich, P-069-1ML), THDOC (Sigma-Aldrich, P2016-5MG), Estriol-16-Glucuronide (Sigma-Aldrich, E1877-10MG), DHEA-S (Sigma-Aldrich, Dehydroepiandrosterone-D5-3-sulfate (DHEAS-D_5_) (2,2,3,4,4,-D_5_) sodium salt solution, D-066-1ML), PE(P-16:0e/0:0) (Avanti Polar lipids, 852470), Androstane-3,17-diol (Sigma-Aldrich, A7755-100MG), 17α-Hydroxyprogesterone (Sigma-Aldrich, H-085-1ML)] and prepared in methanol, except PE(P-16:0e/0:0), which was prepared in chloroform/methanol (8:2).

#### MS acquisition

Metabolic extracts were analyzed by reversed-phase liquid chromatographic (RPLC)-mass spectrometry (MS) in both positive and negative ionization modes. Thermo Q Exactive Hybrid Quadrupole-Orbitrap plus and Q Exactive mass spectrometers (Xcalibur, Thermo Scientific, San Jose, CA, USA) were operated in full MS-scan mode for data acquisition (acquisition from m/z 500 to 2,000) with a scan rate of approximately 4 Hz and a resolution set at 30,000 (at m/z 400). The MS/MS spectra of the QC sample were acquired under different fragmentation energy (25 NCE and 50 NCE) of the top 10 parent ions. The resulting mass spectra were exported into Progenesis QI Software (Nonlinear Dynamics, Durham, NC, USA) for further processing.

#### Chromatographic conditions

RPLC separation was performed using Zorbax SB columns (2.1 X 50mm, 1.8 Micron, 600 Bar; 827700-914) purchased from Agilent Technologies (Santa Clara, CA, USA). Mobile phases for RPLC consisted of 0.06% acetic acid in water (phase A) and 0.06% acetic acid in MeOH (phase B). Metabolites were eluted from the column at a flow rate of 0.6 mL/min, leading to a backpressure of 220–280 bar at 99% phase A. A linear 1%–80% phase B gradient was applied over 9–10 min. The oven temperature was set to 60°C, and the sample injection volume was 5 μL.

### Quantification and Statistical Analysis

#### Section 1: Metabolomics Data Processing

Metabolomic features were extracted with a unique mass/charge ratio and retention time, then aligned and quantified with the Progenesis QI software (Nonlinear Dynamics, Durham, NC, USA, http://www.nonlinear.com/progenesis/qi/). Peak deconvolution was performed under default settings in Progenesis QI. Acquired data were processed using an analysis pipeline written in R (https://www.R-project.org). Progenesis QI output was then processed by removing all metabolites that were quantified in less than 30% of the samples or had a median intensity of less than twofold signal over the noise threshold (S/N < 2). The noise threshold was estimated by using the median signal across all the blank runs (if no quantitation was reported in any of the blank runs, the feature was also included in the analysis, as it likely had good S/N characteristics). Then the data were log-transformed and normalized. For each run, the median of all features was centered to correct for variation in the sample amount. Then for each analyte, a linear correction was applied per batch to correct for any linear decrease or increase in abundance during the acquisition of a batch. In short, for each analyte and each batch, a linear model was fitted with the log-abundance of the analyte as the dependent variable and the acquisition number [run order (randomized)] as the independent variable. The model prediction was interpreted as an underlying drift in mass spectrometric sensitivity and subtracted from the analyte level to yield within-batch normalized abundances. Finally, for each analyte, the abundances were median centered by batch to correct for sensitivity differences between batches. The positive- and negative-mode features were then concatenated for downstream analysis. In total, 9,651 features were included in the final analysis. In addition, for samples with more than 50% of the values missing, the sample was removed (one sample in total). The remaining missing values were imputed by the nearest 10 neighbors using the k-Nearest Neighbor algorithm (Altman, 1992). Note that Discovery and Test Set 1 were normalized together, while samples of Test Set 2 were normalized independently.

We applied principal component analysis (PCA) to examine the overall distribution of the sample data (with all 9,651 features) and check the run quality. The gestational ages (based on first-trimester ultrasound measurements) were superimposed to facilitate the analysis. During the analysis, the vast majority of the samples were separated by pre- and postpartum in PCA space defined by two components, which explained the largest variations (PC1 and 2, Figure 1B), while two samples of a same subject (last two in her collection, before and after childbirth) displayed irregular behavior in PCA and unsupervised clustering analysis. The two samples were treated as outliers and excluded from further analysis. We also performed partial least-squares discriminant analysis (PLS-DA) according to the categories of gestational age (by the mixOmics package).

#### Section 2: Metabolic Features Identification

Metabolite identification was performed using a two-step approach. First, to identify compounds, we used our in-house metabolite library, which contains chemical standards and a manually curated compound list based on accurate mass (m/z, ± 5 ppm), retention time and spectral patterns. Second, further metabolites were identified based on accurate mass, isotope pattern and MS/MS spectra against public databases, including HMDB, MoNA, MassBank, METLIN, and NIST.

Specifically, tandem mass spectrometry (MS/MS) data of QC samples were acquired using a Thermo Q Exactive plus mass spectrometers. The raw MS data (.raw format) were converted to .mgf format files using ProteoWizard (Chambers et al., 2012) (Version 3.0.19095-938eda31a, http://proteowizard.sourceforge.net). Using the metabolic features table (from Waters Progenesis QI) and QC MS/MS data (.mgf format), the metabolic features and MS/MS spectra were matched according to their accurate masses (±25 ppm), and RT values (±30 s) (Shen et al., 2019). If one metabolic feature matched multiple MS/MS spectra, then all matched MS/MS spectra were used for the identification.

Next, the generated MS1/MS2 pairs were automatically searched in the public databases: HMDB (http://www.hmdb.ca/), MoNA (http://mona.fiehnlab.ucdavis.edu/), and MassBank (http://www.massbank.jp/). The MS/MS spectra similarity score was calculated using the forward dot-product algorithm (Stein and Scott, 1994), which considers both fragments and intensities. The similarity score cutoff was set as 0.5.

Furthermore, the metabolic features with MS/MS spectra and not matched in download public databases were searched in the online public databases, METLIN (https://metlin.scripps.edu) and NIST (https://www.nist.gov/). Then the MS/MS spectra match was manually checked to confirm the identifications, which was considered a level 2 identification according to MSI (Viant et al., 2017). In addition, the metabolic peaks with MS/MS spectra that were not matched in public databases were analyzed by MetDNA (Shen et al., 2019) and given a MSI level 4 identification.

Finally, predictors from the machine-learning models were further confirmed with chemical standards by matching the accurate masses (±5 ppm), retention time (±30 s), and the MS/MS spectra for a MSI level 1 identification (Viant et al., 2017).

In the rare cases, when a given metabolic feature was matched differently between different matching methods, we choose the matching based on the identification level: standards > MS/MS > MetDNA.

#### Section 3: Identify Significantly Altered Features/Compounds

A statistical method specialized for multi-testing, SAM (Significance Analysis of Microarrays) (Tusher et al., 2001) was applied to identify metabolic features/compounds altered significantly in metabolome-wide analysis. Specifically, we used SAM to examine the correlation between abundance of each compounds and the gestational age of each sample in Discovery and Test Set 1 cohorts. For all SAM analyses, distribution-independent ranking tests (based on the Wilcoxon test) and the sample-wise permutation (default by the samr package) were used to ascertain significance (false discovery rate, FDR < 0.05). The adjusted gestational ages were included in a number of plots to present the changes in metabolites among individuals, which were calculated by scaling all delivery event timing to 40 weeks. The populational baseline was calculated by taking the mean intensity values of all women with samples before 14 (20 out of 30 women).

To identify top changed compounds with abundance increases or decreases more than 50% during the whole pregnancy (40 weeks), we performed a linear regression between log_2_ abundance and the gestational weeks of samples, and only those compounds with absolute slope larger than log_2_(1.5)/40 weeks = 0.015 were chosen.

#### Section 4: Regularized Partial Correlation Network

The regularized partial correlation network captures the remaining association between two nodes after controlling all other information (indirect correlations) in the network (Epskamp and Fried, 2018). Namely, each node represents a compound, and each edge represents the strength of partial correlation between two nodes after conditioning on all other variables in the datasets. Edge weights represent the partial correlation coefficeients. Lasso (least absolute shrinkage and selection operator) was used to shrink small association coefficient to zero and thus limit spurious correlations in the network. To perform the lasso-based regularized partial correlation, we used qgraph package in R. The tuning parameter gamma(γ), which controls the complexity of the network, was set to 0.5 as suggested (Epskamp and Fried, 2018). Three measures, strength (the sum of absolute edge weight connected to each metabolite), closeness (inverse of the sum of distances from one metabolite to all others), and betweenness (how often one metabolite is in the shortest paths between other metabolites), indicated how important metabolites are in the network.

#### Section 5: Pathway Analysis

The compounds identified by the methods mentioned above were pooled together. We utilized MetaboAnalystR (Chong et al., 2018) (https://github.com/xia-lab/MetaboAnalystR) to perform the metabolite set enrichment analysis (MSEA) (Xia and Wishart, 2010b) as well as metabolic pathway analysis (MetPA) (Xia and Wishart, 2010a) on all identified metabolites. For the potential location/organ analysis on metabolites, we excluded male organ/cell types for MSEA. To quantify pathway activity, we averaged the intensities of all identified metabolites for each pathway that includes no less than three identified metabolites and plotted them on the heatmap (Figure 3B). The pathway activity before 14 weeks were averaged across all available samples and subtracted from all later time points. The statistical significance of the changes in a pathway’s activity across pregnancy was evaluated by global testing (Goeman and Bühlmann, 2007), the default method used by MetaboAnalystR. The topological pathway impacts were quantified using published method (Xia and Wishart, 2010a), with MetaboAnalystR. Human desease states that correlated with pregnancy-related metabolites were calculated based on published metabolomics data (Chong et al., 2018).

#### Section 6: Machine Learning for Pregnancy Timing

Three cohorts of data collected and run at different years but from the same center were used to establish Discovery (subjects N = 21, samples n = 507), Test Set 1 (subjects N = 9, samples n = 245), and Test Set 2 (subjects N = 8, samples n = 32) datasets, excluding non-pregnant (postpartum) samples. We applied lasso (R package: glmnet) in the Discovery dataset to select compounds/metabolic features to build the linear regression model to predict gestational age. A 10-fold cross validation was performed to choose optimal lambda (penalty for the number of features), which determines the performance of the lasso model (number of features included in the model and prediction deviations). For the practical utility of a signature in potential clinical settings, in the identified compound-prediction models, if the number of predictors exceeded five under a given optimal lambda, we increased the lambda value so that the number of predictors is no more than five in the final models. We then used two different methods to evaluate the prediction deviation of our lasso model produced under a given lambda value: 1) A 10-fold cross validation within the Discovery cohort, in which the optimal lambda was used (Baumann and Baumann, 2014, Mayr et al., 2018). In the CV, samples were distributed into folds by subject instead of by samples to prevent person-specific information cross-over between the training folds and the test fold. 2) Validation tests in the separate Test Set 1 and Test Set 2 cohorts, in which independent subjects were included and samples were analyzed one year and three years from the Discovery cohort. We built the model using the optimized lambda and full discovery datasets. This model was applied to the validation cohort for prediction and verification. A linear fitting from the two above evaluations were performed, between the predicted value and the actual values, with Pearson correlation coefficient (R), R^2^, and RMSE reported. The contribution of each predictor (metabolite) in each prediction model is defined by:

$$\text{Contribution}=\text{abs}\left({\text{coefficient}}_{\text{i}}\right)/\text{sum}\left(\text{abs}\left({\text{coefficient}}_{\text{i}}\right)\right)$$

i: the metabolite included in the linear model

Unlike cross-validation in the discovery dataset, validation tests are not prone to hyperparametric selection bias for lambda value. Since samples from Test Set 2 cohort were normalized independently from other samples, a scaling was done in the end. Note that since the sequential nature of the data were not used in the machine-learning methods, other statistical tools, such as recurrent neural networks (e.g., LSTM (Hochreiter and Schmidhuber, 1997, Mayr et al., 2018)), may be explored to improve the model.

For samples collected after 28 weeks (third-trimester samples), we started with 264 level 1 and 2 compounds, and we used a similar discovery and validation pipeline described for predicting gestational age (above) to build logistic regression models predicting the categorical labels of gestational age > 32 and 37 weeks or delivery within 2, 4, 8 weeks. The prediction models for > 20, 24, and 28 weeks were built using samples from all three trimesters. For the prediction on delivery within 2, 4, and 8 weeks, only the 18 women (out of 30) with natural labor onset were included, excluding subjects with induction before labor onset and scheduled cesarean-section (induction by oxytocin/membrane strip after the onset is allowed). To estimate the confidence interval for each AUROC, we performed bootstrapping by person (instead of by samples) for 1000 times, and calculate the 95% confidence interval for AUROC.

#### Section 7: Analyze the discrepancies between metabolic clock (GA prediction model) and first-trimester ultrasound estimations

We evaluated the individual correlation between the predictions made by the metabolic clock and the estimations from first-trimester ultrasound: We first examined the correlation between metabolic clock predictions and the gestational age based on first-trimester ultrasound in individual persons. Each correlation was evaluated by Pearson’s correlation. We then performed meta-analysis across the persons to generate a summary p value, using Fisher’s method, to describe the overall correlation in each cohort (cross-validation in the Discovery, Independent validation of Test Set 1).

Previous literature (Donahue et al., 2010) and our own observations suggest that birth weight and gestational length are positively correlated; later delivery is associated with a heavier absolute birth weight of an infant. To determine whether an infant’s birth weight falls above or below the group mean, we performed a linear regression between the two parameters and took the residuals to represent the birth weight deviation adjusted for delivery timing.

Average Δ(GA_metabolic_- GA_ultrasound_): For each person, at each time point, we examined the differences between the metabolic clock and first-trimester ultrasound estimation of gestational age. These values were averaged for each person to represent the overall relative pace of metabolic clock compared to the first-trimester ultrasound estimation. We then examined the correlation between delivery timing adjusted birth weights and average Δ(GA_metabolomic_- GA_ultrasound_) (Figure 5C).

To examine whether an accelerated metabolic clock (compared to the first-trimester ultrasound estimation) associates with advanced delivery, we performed the correlation between average Δ(GA_metabolomic_- GA_ultrasound_) and delivery timing, only in women with a natural labor onset (Figure 5D).

## Supplemental Information

Document S1. Tables S1, S2, and S4

Table S3 The 264 identified level-1 and level-2 metabolites that have HMDB IDs, and their log_2_(intensity) used for the machine-learning model, Related to Figure 4
