# Supplementary material for: Metabolic Dynamics and Prediction of Gestational Age and Time to Delivery in Pregnant Women
Source: Cell. 2020 Jun 25;181(7):1680–1692.e15. doi: 10.1016/j.cell.2020.05.002 (PMC7327522; doi:10.1016/j.cell.2020.05.002)
Supplement: Document S1. Tables S1, S2, and S4 [file mmc1.pdf]

Cell, Volume *181*

Supplemental Information

Metabolic Dynamics and Prediction of Gestational Age and Time to Delivery in Pregnant Women

Liang Liang, Marie-Louise Hee Rasmussen, Brian Piening, Xiaotao Shen, Songjie Chen, Hannes Röst, John K. Snyder, Robert Tibshirani, Line Skotte, Norman CY. Lee, Kévin Contrepois, Bjarke Feenstra, Hanyah Zackriah, Michael Snyder, and Mads Melbye

Supplemental Tables

Table S1. Information of the list of the top identified metabolites significantly associated with pregnancy progression (FDR < 0.05). Related to Figure 2

Metabolites	HMDB ID	Pathways	Weekly log2(intensity) Changes
Estriol-16-Glucuronide	HMDB06766	Steroid hormone biosynthesis	0.1674
Estrone 3-sulfate	HMDB01425	Steroid hormone biosynthesis	0.0879
Tetrahydrodeoxycorticosterone (THDOC)	HMDB00879	Steroid hormone biosynthesis	0.0755
N-Acetyl-D-glucosamine	HMDB00215	Others	0.0619
Progesterone	HMDB01830	Steroid hormone biosynthesis	0.0617
17α-Hydroxyprogesterone	HMDB00374	Steroid hormone biosynthesis	0.0586
Caffeine	HMDB01847	Caffeine metabolism	0.0459
3-Acetoxypyridine	HMDB33131	Others	0.0446
5-Pregnane-3,17-diol-20-one 3-sulfate	METPA0001	Steroid hormone biosynthesis	0.0415
7alpha,24-Dihydroxy-4-cholesten-3-one	HMDB12457	Bile acid biosynthesis	0.0404
Theophylline	HMDB01889	Caffeine metabolism	0.0378
1-Methylxanthine	HMDB10738	Caffeine metabolism	0.0357
C16 PAF (Platelet-activating factor)	HMDB39527	Fatty acid metabolism	0.0326
Cortisone	HMDB02802	Steroid hormone biosynthesis	0.0314
Docosadienoic acid	HMDB61714	Fatty acid metabolism	0.0312
Tetracosapentaenoic acid	HMDB06322	Fatty acid metabolism	0.0298
Pregnenolone sulfate	HMDB00774	Steroid hormone biosynthesis	0.0264
Theobromine	HMDB02825	Caffeine metabolism	0.0261
Cortisol	HMDB00063	Steroid hormone biosynthesis	0.0260
17,18-EpETE	HMDB10212	Fatty acid metabolism	0.0253
Corticosterone	HMDB01547	Steroid hormone biosynthesis	0.0239
Androstane-3,17-diol	HMDB00495	Steroid hormone biosynthesis	0.0237
Taurochenodeoxycholate	HMDB00951	Bile acid biosynthesis	0.0234
Erucic acid	HMDB02068	Fatty acid metabolism	0.0220
Sphingosine	HMDB00252	Others	0.0205
Dodecanoylcarnitine	HMDB02250	Fatty acid metabolism	0.0197

Tetracosatetraenoic acid	HMDB06246	Fatty acid metabolism	0.0190
Tetracosahexaenoic acid	HMDB02007	Fatty acid metabolism	0.0186
7-Methylguanine	HMDB00897	Others	0.0179
Cyclo(leucylprolyl)	HMDB34276	Caffeine metabolism	0.0169
PC(18:1(9Z)e/2:0)	HMDB11148	Phospholipid metabolism	-0.0151
LPE(20:3)	HMDB11484	Phospholipid metabolism	-0.0156
Ketoisovaleric acid	HMDB00019	Amino acid metabolism	-0.0160
Valylhistidine	METPA0001	Amino acid metabolism	-0.0161
Tricosanoic acid	HMDB01160	Fatty acid metabolism	-0.0165
Androsterone sulfate	HMDB02759	Steroid hormone biosynthesis	-0.0166
MG(18:1)	HMDB11536	Fatty acid metabolism	-0.0169
PC(22:1/22:1) (Lecithin)	HMDB14291	Phospholipid metabolism	-0.0170
3-Hydroxyoleylcarnitine	HMDB13132	Fatty acid metabolism	-0.0170
Hexadecadienoylcarnitine	HMDB13334	Fatty acid metabolism	-0.0170
Sinapyl alcohol	HMDB13070	Others	-0.0186
Hydroxybupropion	HMDB12235	Others	-0.0188
2-Phenylbutyric acid	HMDB00329	Others	-0.0191
Glycochenodeoxycholate	HMDB00637	Bile acid biosynthesis	-0.0192
LPC(18:2)	HMDB10386	Phospholipid metabolism	-0.0194
LPE(22:4)	HMDB11493	Phospholipid metabolism	-0.0196
LPC(24:0)	HMDB10405	Phospholipid metabolism	-0.0203
LPE(20:1)	HMDB11482	Phospholipid metabolism	-0.0208
Isobutyryl-L-carnitine	HMDB00736	Fatty acid metabolism	-0.0213
LPE(22:1)	HMDB11491	Phospholipid metabolism	-0.0213
Glycyrrhetinic acid	HMDB11628	Fatty acid metabolism	-0.0218
LPE(20:0)	HMDB11481	Phospholipid metabolism	-0.0222
8,9-DHET	HMDB02311	Fatty acid metabolism	-0.0240
LPC(P-18:0)	HMDB13122	Phospholipid metabolism	-0.0244
LPC(20:5)	HMDB10397	Phospholipid metabolism	-0.0248
MG(14:1)	HMDB11531	Fatty acid metabolism	-0.0273
LPC(P-18:1)	HMDB10408	Phospholipid metabolism	-0.0280
PE(P-16:0e/0:0)	HMDB11152	Phospholipid metabolism	-0.0285
Dehydroepiandrosterone sulfate (DHEA-S)	HMDB01032	Steroid hormone biosynthesis	-0.0294
Oleoylcarnitine	HMDB05065	Fatty acid metabolism	-0.0312
beta-Glycyrrhetinic acid	HMDB34517	Fatty acid metabolism	-0.0319
LPC(17:0)	HMDB12108	Phospholipid metabolism	-0.0328
LPC(P-16:0)	HMDB10407	Phospholipid metabolism	-0.0332
MG(24:1)	HMDB11559	Fatty acid metabolism	-0.0334

MG(24:0)	HMDB11558	Fatty acid metabolism	-0.0346
LPE(22:2)	HMDB11492	Phospholipid metabolism	-0.0440
MG(22:2)	HMDB11553	Fatty acid metabolism	-0.0466
MG(20:0)	HMDB11542	Fatty acid metabolism	-0.0483

Table S2. Summary of the metabolic features selected by machine learning in the gestational age prediction model. Related to Figure 4

Fragments formulae are given for exact mass of ions detected.

Metabolic feature	Contribution	m/z	RT/min	Polarity	Compound/Ion Formula	MSI Confidence Level[23]
M1	0.15640	399.1482	6.70	negative	C19H28O7S	3
M2	0.10627	438.2974	9.52	positive	PE(P-16:0e/0:0)	1
M3	0.07564	413.3057	10.94	negative	C27H42O3	3
M4*	0.06705	529.2412	8.02	positive	C29H38O9Na	3
M5	0.05015	510.9290	5.38	positive		
M6	0.04615	417.3354	10.48	positive	C27H44O3	3
M7	0.04528	531.2562	7.73	positive	C27H40O9Na	4
M8	0.04311	511.2904	7.82	negative	C27H44O9	3
M9	0.03431	399.1481	5.71	negative	C19H28O7S	3
M10#	0.03074	257.2261	8.33	positive	C19H29	3
M11	0.02803	315.2314	7.58	positive	C21H30O2	3
M12	0.02468	519.2564	8.64	positive		
M13	0.02443	563.1797	6.59	positive		
M14	0.02437	463.1968	6.66	negative	Estriol-16-Glucuronide	1
M15*	0.02206	353.2085	7.91	positive	C21H30O3Na	3
M16*	0.01911	487.1935	6.61	positive	C24H32O9Na	3
M17	0.01820	483.2593	8.33	negative	C25H40O9	4
M18	0.01673	431.3153	9.78	negative	C27H44O4	4
M19	0.01412	415.3192	9.47	positive	C27H42O3	3
M20#	0.01353	301.2522	7.74	positive	C21H33O	3
M21#	0.01333	331.2265	7.92	positive	C21H31O3	3
M22	0.01276	538.3501	8.88	positive	C26H52NO8P	4
M23	0.01265	493.2799	9.13	negative	C27H42O8	4
M24	0.01202	263.1388	1.89	positive	Prolylphenylalanine	2
M25	0.01188	371.1884	9.70	negative	C19H31O5S	3
M26	0.00971	465.3449	7.39	positive	C23H49N2O5P or C30H50O8	4
M27#	0.00928	297.2210	7.42	positive	C21H29O	4
M28	0.00920	593.3692	10.17	negative	C33H54O9	3
M29	0.00900	347.2591	9.52	negative	C22H36O3	4
M30	0.00768	498.3037	9.26	negative		4
M31#	0.00616	319.1648	2.58	positive	C21H31O3	4
M32	0.00601	401.1638	7.89	negative	C19H30O7S	3

M33	0.00584	525.2694	6.33	negative	C27H42O10	3
M34	0.00506	381.1011	5.39	negative	C18H22O7S, likely 6-Ketoestriol sulfate	3
M35	0.00356	315.2315	9.30	positive	Progesterone	1
M36	0.00175	821.2961	9.13	negative		
M37	0.00125	527.2852	8.26	negative	C27H44O10	3
M38	0.00089	653.2862	9.26	negative		
M39	0.00082	798.3566	8.45	positive		
M40	0.00032	260.1064	9.78	negative		
M41	0.00027	823.3115	9.26	negative		
M42[§]	0.00020	337.2135	9.30	positive	C21H30O2Na	3

* Features are sodium ionized.

[#] Features are fragments.

[§] The feature is sodium ionized progesterone.

Table S4. Information of the seven predictors in the machine-learning models. Related to Figure 4 and Figure 5

Polarity	m/z	RT (second)	Compound Name	Formula	Adduct	m/z Error (ppm)	RT Error (s)	MS/MS Similarity Score (Dot Product)
Positive	299.2365	526.8	THDOC (3α,21-Dihydroxy-5α-pregnan-20-one)	$C_{21}H_{34}O_3$	$[M+H-2H_2O]^-$	3.31	15.6	0.75
Negative	463.1968	413.5	Estriol-16-Glucuronide	$C_{24}H_{32}O_9$	$[M-H]^-$	0.02	1.2	0.88
Positive	315.2315	574.9	Progesterone	$C_{21}H_{30}O_2$	$[M+H]^+$	2.87	1.2	0.99
Positive	438.2974	555.5	PE(P-16:0e/0:0)	$C_{21}H_{44}NO_6P$	$[M+H]^+$	2.40	0.6	0.99
Negative	367.1583	572.3	Dehydroepiandrosterone sulfate (DHEA-S)	$C_{19}H_{28}O_5S$	$[M-H]^-$	1.04	9.0	0.83
Positive	257.2260	548.0	Androstane-3,17-diol	$C_{19}H_{32}O_2$	$[M+H-2H_2O]^-$	3.60	2.4	0.87
Positive	331.2262	496.7	17α-Hydroxyprogesterone	$C_{21}H_{30}O_3$	$[M+H]^+$	3.38	29.4	0.98
